# *KRAS* G12V Mutation Detection by Droplet Digital PCR in Circulating Cell-Free DNA of Colorectal Cancer Patients

**DOI:** 10.3390/ijms17040484

**Published:** 2016-04-01

**Authors:** Susana Olmedillas López, Dolores C. García-Olmo, Mariano García-Arranz, Héctor Guadalajara, Carlos Pastor, Damián García-Olmo

**Affiliations:** 1 Health Research Institute-Fundación Jiménez Díaz University Hospital (IIS-FJD), Madrid 28040, Spain; mariano.garcia@fjd.es (M.G.-A.); damian.garcia@uam.es (D.G.-O.); 2 Experimental Research Unit, General University Hospital of Albacete, Albacete 02006, Spain; doloresg@sescam.jccm.es; 3 Department of Surgery, School of Medicine, Autónoma University of Madrid, Madrid 28029, Spain; h.guadalajara@gmail.com; 4 Department of General Surgery, General Hospital of Villalba, Madrid 28400, Spain; 5 Department of Surgery, Fundación Jiménez Díaz University Hospital, Madrid 28040, Spain; cpastorid@fjd.es

**Keywords:** *KRAS*, colorectal cancer, plasma, droplet digital PCR, circulating cell-free DNA

## Abstract

*KRAS* mutations are responsible for resistance to anti-epidermal growth factor receptor (EGFR) therapy in colorectal cancer patients. These mutations sometimes appear once treatment has started. Detection of *KRAS* mutations in circulating cell-free DNA in plasma (“liquid biopsy”) by droplet digital PCR (ddPCR) has emerged as a very sensitive and promising alternative to serial biopsies for disease monitoring. In this study, *KRAS* G12V mutation was analyzed by ddPCR in plasma DNA from 10 colorectal cancer patients and compared to six healthy donors. The percentage of *KRAS* G12V mutation relative to wild-type sequences in tumor-derived DNA was also determined. *KRAS* G12V mutation circulating in plasma was detected in 9 of 10 colorectal cancer patients whose tumors were also mutated. Colorectal cancer patients had 35.62 copies of mutated *KRAS*/mL plasma, whereas in healthy controls only residual copies were found (0.62 copies/mL, *p* = 0.0066). Interestingly, patients with metastatic disease showed a significantly higher number of mutant copies than M0 patients (126.25 *versus* 9.37 copies/mL, *p* = 0.0286). Wild-type *KRAS* was also significantly elevated in colorectal cancer patients compared to healthy controls (7718.8 *versus* 481.25 copies/mL, *p* = 0.0002). In conclusion, *KRAS* G12V mutation is detectable in plasma of colorectal cancer patients by ddPCR and could be used as a non-invasive biomarker.

## 1. Introduction

In the past few years, cancer treatment has evolved markedly towards more personalized targeted therapies. Metastatic colorectal cancer treatment frequently combines surgical resection with adjuvant therapies that include monoclonal antibodies, such as EGFR-targeted antibodies (cetuximab and panitumumab). However, only patients with *KRAS* wild-type tumors can benefit from anti-epidermal growth factor receptor (EGFR) therapies, since it has been demonstrated that *KRAS* mutations predispose to drug resistance [1]. Thus, tumor genotyping becomes crucial to decisions on clinical treatment. However, secondary resistance could appear as a result of intratumoral heterogeneity, clonal evolution and selection, *i.e.*, subpopulations of tumor cells that become resistant to treatment and proliferate [2]. Molecular analysis is routinely performed using DNA extracted from tissue, but taking serial biopsies entails many difficulties and is not always possible due to several factors: Tumors or metastases not accessible for biopsy, insufficient material available for genotyping, discomfort for the patient, risk of tumor spread due to the procedure itself, potential clinical complications, economic considerations, difficulties in acquiring samples from different medical centers, and/or treatment contraindications [3,4]. We should be able to overcome these issues and rapidly identify biomarkers in a cost-effective and non-invasive manner to monitor treatment response at different time points during the course of disease. To this end, DNA fragments released by tumor cells, which can be found circulating in plasma and are termed as circulating tumor DNA (ctDNA), have given rise to the concept of “liquid biopsy” [2]. The main problem impeding more widespread use of liquid biopsy is that certain clinical scenarios exist, particularly at early stages of disease, where levels of circulating tumor DNA are below the limits of detection of currently applied techniques [3].

In recent years, many efforts have been made to develop highly specific and sensitive techniques for detection of low-abundance *KRAS* mutations, including real-time PCR, coamplification at lower denaturation temperature-PCR (COLD-PCR), pyrosequencing, or digital PCR [5,6]. Nowadays, digital PCR has become one of the mainstream methodologies for rare mutation detection, but this partition-based technique is actually not new. The term “digital PCR” was coined and described in 1999 by Vogelstein *et al.* [7] in a study aimed at detecting a variant of a single-nucleotide polymorphism of the *RAS* oncogene in samples where wild-type sequences were predominant. Indeed, in the previous decade, this method was used under the names “single molecule PCR” or “limiting dilution PCR” (reviewed in [8]). However, the results of the first digital PCR studies were limited by technical and economic hurdles, and it was not until the development of new instrumentation based on nanofluidics and emulsion chemistries that this technology has become more affordable and available for routine implementation [9].

Droplet digital PCR (ddPCR) technology performs a water-in-oil emulsion of the PCR reaction mixture, which allows for massive sub-partitioning into hundreds to millions of independent reactions, creating a synthetic enrichment effect that dramatically increases the capability of detecting rare mutations present at very low levels in the sample [10]. After amplification in a thermal cycler, the number of positive partitions (where the amplified target sequence is detected) and negative partitions (in which there is no signal of amplification), are counted as a binary or “digital” system. A Poisson correction is then applied for quantification of the mean number of target sequences per partition [11].

Several platforms of ddPCR have been developed by different manufacturers, such as Fluidigm, Sysmex Inostics (BEAMing Digital PCR), Bio-Rad Laboratories, or RainDance Technologies. Some of them have already been tested for detection of *KRAS* mutations producing different results [12,13,14,15,16,17,18,19,20]. The present study is aimed at evaluating the sensitivity and reproducibility of a new droplet digital PCR system (Bio-Rad QX-200 platform) for detection of *KRAS* G12V mutation in samples of plasma where this mutation has previously been confirmed. This particular mutation was chosen because it has been associated with a worse progression in our series of patients, showing a markedly poor clinical outcome, high rate of post-operative complications, and short time of survival.

## 2. Results

The human adenocarcinoma cell line SW480, which harbors the *KRAS* G12V mutation in homozygosis, was used to assess the analytical sensitivity of the assay. We performed serial dilutions of DNA from the SW480 cell line (from 5 to 12.5 pg/µL) into a constant background of wild-type genomic DNA from leukocytes (130 ng per well). Non-diluted cell line-derived DNA showed a fractional abundance of 99.99% of mutant DNA, with a residual presence of wild-type copies. *KRAS* G12V mutation could be detected even at a dilution of 1/4000, which corresponds to a fractional abundance of 0.025%, maintaining the linearity of the assay (*R*^2^ = 0.998).

We only detected wild-type *KRAS* sequences and no mutant copies in 50 ng/µL DNA extracted from healthy donor leukocytes (*n* = 4). Background from water added to the reaction mixture instead of DNA was also analyzed (*n* = 4) and no mutant copies were detectable, although a limited number of positive events for wild-type sequences were found.

In DNA extracted from fresh-frozen portions of tumor mucosa, the percentage of *KRAS* G12V mutation relative to wild-type sequences was 36.83% (*n* = 10, median).

We analyzed the presence of *KRAS* G12V mutation in the plasma of six healthy donors. In three of these donors, we detected very low concentrations, between 1.25 and 1.87 copies/mL of plasma. In the other three, only wild-type sequences of *KRAS* were detected.

*KRAS* G12V mutation was detected in all colorectal cancer patients tested, with the exception of one. This sample was obtained from a patient with a tumor staged as T1N0M0 (Table 1). Colorectal cancer patients had 35.62 copies of mutated *KRAS* per milliliter of plasma (median), whereas in healthy controls only residual copies were found (0.62 copies/mL, *p* = 0.0066). Patients with metastatic disease showed a significantly higher number of mutant copies/mL plasma than M0 patients (median, 126.25 and 9.37 copies/mL, respectively, *p* = 0.0286).

The amount of wild-type *KRAS* circulating in plasma was also significantly elevated in colorectal cancer patients in comparison to healthy controls (median, 7718.8 *versus* 481.25 copies/mL, respectively, *p* = 0.0002).

## 3. Discussion

*KRAS* mutations have become routinely used as molecular biomarkers in clinical practice for management and monitoring of metastatic colorectal cancer patients [21]. Given the fact that patients carrying *KRAS* mutations do not respond to anti-EGFR antibodies (cetuximab and panitumumab), a precise, sensitive, and specific method of *KRAS* genotyping is essential for decision making. Furthermore, mutational status of *KRAS* should be determined not only at diagnosis, but also during treatment follow-up. Point mutations often appear as a result of intratumoral heterogeneity and clonal selection in primary tumors and/or metastases as they evolve during disease progression, leading to the development of secondary drug resistance [2]. Determination of *KRAS* mutations in plasma is based on the presence of circulating tumor DNA (ctDNA) and offers the possibility of constant monitoring without the need for serial biopsies. This alternative source of DNA for tumor genotyping is termed “liquid biopsy” and has gained increasing interest because a blood draw is less invasive, faster, and more feasible than tissue sampling [3].

Tumors from patients enrolled in this study were previously proven to carry *KRAS* G12V mutation as determined by Sanger sequencing from fresh-frozen tumors. Of all the most common *KRAS* codon 12 mutations, we chose G12V because it has been related to a more aggressive phenotype and worse progression of the disease [22,23,24,25,26,27]. In fact, the cohort of *KRAS* G12V patients that we included in this study was remarkable because of its poor clinical outcome, high rate of post-operative complications, and short time of survival (Table 1).

The main shortcoming of our study was the limited number of mutation-carrying patients available for analysis (*n* = 10). Although the G12V mutation is considered to be one of the most frequent *KRAS* codon 12 mutations in colorectal cancer patients—particularly in those with liver metastasis (ranging from 20.5% to 32.8%) [21,27]—the number of *KRAS* G12V-mutated samples in our population of study at La Paz University Hospital was very low (13 out of 554 total patients analyzed, representing 15.12% of total *KRAS* codon 12 mutations).

Our aim was to evaluate the sensitivity and reproducibility of a new droplet digital PCR platform for detection of previously confirmed *KRAS* G12V mutation in plasma samples.

We achieved high analytical sensitivity in the assay, reaching 1/4000 dilution, corresponding to a fractional abundance of 0.025%. Sanmamed *et al.* [28] recently reported a higher sensitivity for the detection of *BRAF* V600E mutation in plasma of melanoma patients using the same ddPCR platform (a fractional abundance of 0.005%). However, their limit of detection was established as 1 copy of mutant DNA/mL. Oxnard *et al.* [16] also reported a detection sensitivity of 5 to 50 mutant copies in a background of 10,000 wild-type copies using serial dilutions of mutant DNA, which corresponds to a prevalence between 0.005% and 0.01%, depending on the mutation assayed (including *EGFR* L858R, *EGFR* exon 19 deletion, and *KRAS* G12C). These discrepancies are probably a consequence of differences in data normalization criteria and/or methodologies for assay sensitivity analysis, such as serial dilution preparation and starting concentration of mutant DNA (e.g., 16 ng/µL in Sanmamed’s article *versus* 5 ng/µL in our study).

Given the fact that positive mutation events were still detectable at 1/4000 dilution, we could have tested further dilutions until the number of counts reached zero. However, background of mutated copies in healthy donors ranged between 1.25 and 1.87 copies/mL of plasma in three of these donors, so we established the positivity threshold at 1.87 copies/mL. Thus, in our study, to be considered as positive for the mutation, plasma samples from colorectal cancer patients had to contain more than 1.87 copies/mL. Other authors used a threshold of 0.5 to 1 copies/mL for a positive result, even when a healthy donor showed 12 copies/mL [28]. In some reports, the threshold varies depending on the mutation assayed and the number of cases correctly identified as positive (from 0.5 to 6 copies/mL) [16]. These differences in thresholds raise awareness about the need for a consensus and the adoption of some guidelines by the scientific community to standardize experimental procedures in ddPCR technology [11].

The ddPCR platform used in this study has shown a strikingly high sensitivity of detection. Comparative studies have shown that ddPCR exceeds other methods, such as real-time PCR or pyrosequencing [29,30,31,32], which are also more expensive, labor-intensive, and require more manipulation, thus increasing the risk of contamination. It has been recently reported that ddPCR can be performed in liquid biopsy samples from breast cancer patients for <€1000 per patient in reagents and <€50 per time point, currently making it much more cost-effective than approaches where whole-genome sequencing is used. In addition, implementation of this analysis in routine clinical management seems feasible with respect to time frame, since a ddPCR test on patient plasma samples can be performed within 1 day [33].

In our study, the percentage of *KRAS* G12V in tumor-derived DNA was 36.83% (*n* = 10, median). This percentage could not be correlated to either the number of copies per mL of plasma or to the presence of metastasis. Intratumoral heterogeneity (*i.e.*, clones or subpopulations of cells with different genetic profiles inside the tumor) and differences in tumor cellularity of biopsies (*i.e.*, the fragment of mucosa that is taken for analysis may not be representative of the whole tumor, introducing a bias due to tumor fragment contamination by normal cells) [20,34] could explain the lack of correlation between the percentage of mutant DNA in the tumor and the rest of the parameters analyzed in this study.

We observed that colorectal cancer patients had mutated *KRAS* circulating in plasma as well as elevated levels of wild-type *KRAS* in comparison to healthy controls (*p* = 0.0066 and *p* = 0.0002, respectively). These results are in agreement with recent reports, where circulating tumor DNA levels showed a quantitative predictive value of poor clinical outcome in melanoma and breast cancer [28,33]. However, elevated levels of wild-type DNA should not be used as cancer biomarkers, since they often rise in many other nonmalignant conditions, such as heart dysfunction, inflammation, pregnancy, heavy smoking, or after exercise or trauma [2,3,35].

It is also noteworthy that patients with metastatic disease showed a significantly higher number of mutant copies/mL plasma than M0 patients (*p* = 0.0286). In line with these results, Bettegowda *et al.* [4] reported that detectable levels of ctDNA were present in 49% to 78% of patients with localized tumors *versus* 86% to 100% of patients with metastatic colon, pancreas, breast, and gastroesophageal tumors. In their study, the concentration of ctDNA in plasma increased with stage and was predictive of a lower survival rate, whereas more than a half of patients with early stages of disease had undetectable levels of ctDNA. Taking these observations into account, it is not surprising that we found one patient in our series with localized stage I disease where we could not detect mutant DNA circulating in plasma. Future studies will be conducted and will include a higher number of T1 patients in order to verify whether levels of mutated copies in circulating cell-free DNA at early stages are too low to be detected by our method.

Reinert *et al.* [35] has recently found a close correlation between the amount of mutant DNA detected by ddPCR in serial post-surgery plasma samples and the clinical disease course. In their retrospective study, circulating tumor DNA levels decreased after tumor resection, radiofrequency ablation, or chemotherapy, but they increased again in the previous months to diagnosis of clinical recurrence. In all relapsing patients, a raise in ctDNA anticipated the appearance of recurrent disease up to 10 months earlier than conventional diagnostic tools, such as computed tomography (CT). However, ctDNA was undetectable in non-relapsing patients. This study highlights the relevance of detecting and quantifying ctDNA by ddPCR during disease follow-up: the procedure provides an early biomarker of recurrence and offers the possibility to evaluate the response to treatment, shortening the time needed to make changes in therapeutic regimens and clinical interventions.

Analysis of molecular markers in ctDNA could also have significant implications for early detection and prevention of disease, identifying pathogenic changes before symptoms develop. Epidemiology research traditionally has focused on risk factors associated with colorectal cancer, whereas molecular pathology has explored the molecular characteristics of tumors involved in carcinogenesis and tumor behavior. These two approaches have now converged in a relatively new field termed “molecular pathological epidemiology (MPE)”, based on the molecular classification of cancer into distinct subtypes [36]. MPE is emerging as a new standard of medicine for intrinsically heterogeneous diseases, especially cancer [37]. The concept of MPE, which was coined by Ogino and Stampfer, consists of multidisciplinary investigation of the relationship between exogenous and endogenous factors of interest for tumor initiation, progression, and response to treatment [36,38]. Among its major applications are molecular biomarker discovery and validation for risk assessment, early detection, diagnosis, and decision making on interventions. By providing a better definition of phenotype, MPE could improve our understanding of etiologic heterogeneity, the relevance of personalized preventive strategies depending on particular risk factors, exposure (dietary, lifestyle, microbial and chemical), host susceptibilities, and individual profiles [37]. Thus, the strategy proposed in this study for the analysis of tumor molecular signatures on liquid biopsy could impact large-scale population studies and contribute to the expansion of the MPE research area.

A growing body of evidence demonstrates the utility of ddPCR, not only to identify specific mutations, but also to monitor disease progression and the emergence of drug resistance in many types of cancers, such as colorectal cancer, melanoma, lung or breast cancer [16,20,28,39]. In fact, it is gaining consideration as one of the most effective methods of measuring minimal residual disease in oncologic conditions, such as melanoma [40], multiple myeloma, mantle cell lymphoma, and follicular lymphoma [31].

To our knowledge, this is the first study to describe the detection of *KRAS* G12V mutation circulating in plasma using this particular droplet digital PCR platform. The reported results come from a limited series of patients which requires further validation, although our data show that *KRAS* G12V mutation is detectable in plasma of colorectal cancer patients using this ddPCR system, and could be useful as a non-invasive biomarker of drug resistance and response to treatment during disease progression.

## 4. Materials and Methods

### 4.1. Cells

We purchased the SW480 human adenocarcinoma cell line from the American Type Culture Collection (ATCC, Manassas, VA, USA). Cells were cultured at 37 °C in a 5% CO_2_ humidified atmosphere with Dulbecco’s Modified Eagle Medium (DMEM, Gibco, Invitrogen, Life Technologies, San Diego, CA, USA) medium supplemented with 10% heat inactivated fetal bovine serum and 1% penicillin/streptomycin.

### 4.2. Patients and Healthy Subjects

We selected 10 patients with colorectal cancer who had undergone resection of primary tumors from 2004 to 2013 in the Department of General Surgery at La Paz University Hospital (PI-138, Madrid, Spain) (*n* = 8) and General University Hospital of Albacete (05/12, Albacete, Spain) (*n* = 2), according to a protocol approved by the Ethics Committee of these institutions. The patients had *KRAS* mutated tumors (G12V) that had been detected by sequencing. In all cases, histopathologic analysis revealed the tumors to be adenocarcinomas.

Six healthy donors were included in the study after informed consent was provided.

### 4.3. Sample Collection

Blood samples were collected in EDTA tubes (9 mL) immediately before surgery. All samples were centrifuged at 1800× *g* for 10 min. Plasma was collected, subjected to a second centrifugation at 3000× *g* for 10 min, aliquoted and stored at −80 °C until analysis.

### 4.4. DNA Extraction

Circulating DNA from plasma and genomic DNA from cells and tumors were extracted with the QIAamp DNeasy Blood and Tissue Kit (Qiagen, Hilden, Germany) according to the manufacturer’s protocol. DNA quantification was performed in a NanoDrop spectrophotometer (Thermo Scientific, Waltham, MA, USA).

### 4.5. Mutation Detection by ddPCR

ddPCR assays were performed with the Prime-PCR™ ddPCR™ Mutation Detection Assay Kit (Bio-Rad, Hercules, CA, USA) using an amplicon of 57 nt. DNA from the SW480 cell line was used as a positive control and DNA from leukocytes of a healthy donor served as a negative control. Background from water added to the reaction mixture instead of DNA was analyzed. This study was performed on a QX200 Droplet Digital PCR System (Bio-Rad), consisting of a C1000 Touch Thermalcycler, a QX200 Droplet Generator, and a QX200 Droplet Reader. The PCR reaction mixture (20 µL) contained 10 µL of ddPCR Supermix (no dUTP) for probes, 1 µL of each primer/probe mix (target and reference, labeled with HEX and FAM fluorophores, respectively), and 8 µL of plasma-extracted DNA. A total amount of 130 ng of DNA was added per well in case of positive and negative controls and in DNA extracted from tumors. The thermal cycling started with 10 min at 95 °C, followed by 40 cycles of 94 °C for 30 s and 55 °C for 60 s. Results were analyzed using Quantasoft v.1.7 software (Bio-Rad) and reported as copies per mL of plasma or % of mutant DNA in the tumor. Four replicates of each sample were analyzed.

### 4.6. Statistical Analyses

All statistical calculations were done using GraphPad InStat software. The Mann-Whitney test for significance was utilized because some of the data were not normally distributed and this test makes no assumption on data distribution.

## 5. Conclusion

*KRAS* G12V mutation is detectable in plasma of colorectal cancer patients by ddPCR and could be used as a non-invasive biomarker of drug resistance during disease progression.

## Figures and Tables

**Table 1:** Clinical features of patients included in the study.

Patient ID	Age (Years)	Sex	TNM	Stage	Survival	*KRAS* G12V (Copies/mL)	Fractional Abundance (%)
53	85	M	T3N0M0	IIa	6 years	7.50	62.79
113	80	F	T1N0M0	I	>2 years	0.00	40.42
118	64	M	T3N2M1 Liver	IV	>2 years	25.00	6.16
130	49	F	T3N1M1 Liver	IV	8 months	197.5	20.60
158	84	M	T4N0M0	IIb	1 year	11.25	34.87
220	69	M	T4N2M1 Bone	IV	2 months	55.00	38.80
257	85	M	T3N0MX	IIa	8 days	46.25	53.20
258	86	M	T3N0MX	IIa	20 days	110.00	33.70
522	77	M	T3N0M0	IIa	18 months	13.75	23.76
532	60	M	T4N1M2 Liver Lung	IV	10 days	2412.5	77.82

This cohort of *KRAS* G12V patients showed a markedly poor clinical outcome, high rate of post-operative complications, and short time of survival.

## Abbreviations

*KRAS*	Kirsten rat sarcoma viral oncogene homolog
*EGFR*	epidermal growth factor receptor
*BRAF*	B-Raf proto-oncogene serine/threonine kinase
DdPCR	droplet digital PCR
CtDNA	circulating tumor DNA
COLD-PCR	coamplification at lower denaturation temperature-PCR
BEAMing	beads, emulsions, amplification, and magnetic
